# Supplementary material for: Early childhood trajectories of domain-specific developmental delay and gestational age at birth: An analysis of the All Our Families cohort
Source: PLoS One. 2023 Dec 27;18(12):e0294522. doi: 10.1371/journal.pone.0294522 (PMC10752539; doi:10.1371/journal.pone.0294522)
Supplement: Attachment — Submitted filename: Response to Reviewers.docx [file pone.0294522.s001.docx]

**RESPONSE TO REVIEWERS**

**JOURNAL REQUIREMENTS:**

1. Please ensure that your manuscript meets PLOS ONE's style requirements, including those for file naming.

**Author response:** We have read the PLOS ONE's style requirements, and have revised the manuscript to ensure this manuscript meets said requirements.

2. Please amend either the title on the online submission form (via Edit Submission) or the title in the manuscript so that they are identical.

**Author response:** We have amended the title on the submission form and manuscript to ensure they are identical.

**ADDITIONAL EDITOR COMMENTS:**

1. It is an interesting study where authors investigated the developmental trajectories in children according to gestational age at birth. However, some technical issues have been raised by the reviewers. Authors are suggested to address them carefully, and improve the manuscript accordingly.

**Author response:** Thank you for the opportunity to improve this manuscript through revision and resubmit to PLOS ONE, we address issues raised by reviewers below.

**REVIEWER'S RESPONSES TO QUESTIONS**

1. Is the manuscript technically sound, and do the data support the conclusions?

Reviewer #1: Yes

Reviewer #2: Yes

Reviewer #3: Partly

2. Has the statistical analysis been performed appropriately and rigorously?

Reviewer #1: Yes

Reviewer #2: I Don't Know

Reviewer #3: Yes

3. Have the authors made all data underlying the findings in their manuscript fully available?

Reviewer #1: No

Reviewer #2: No

Reviewer #3: Yes

**Author response:** Reviewers #1 and #2 noted that all data underlying the findings in the present manuscript was not made fully available. As we note within the data availability statement, data cannot be shared publicly because of legal and ethical agreements.

Data that support the findings of this study are available upon request from the All Our Families Cohort (<https://allourfamiliesstudy.com/data-access/>). Requests must undergo ethical review before entering into a data-sharing agreement with the institution to protect the privacy and confidentiality of the cohort’s participants. The metadata of variables used within this study can be found at <https://www.maelstrom-research.org/study/aob>.

As per PLOS ONE’s “Acceptable Data Access Restrictions”, we have described these restrictions within the “Data availability statement” as well as the “Participant consent and ethics statement” section of the manuscript (lines 107-114 within the original or 112-118 after revisions). We hope that Reviewers #1 and #2 are amenable to this point on data sharing, as we are unable to make the underlying data set publicly available for both legal and ethical reasons.

4. Is the manuscript presented in an intelligible fashion and written in standard English?

Reviewer #1: Yes

Reviewer #2: Yes

Reviewer #3: Yes

**REVIEWER COMMENTS TO THE AUTHOR:**

**Reviewer #1:**

1. The authors have provided a well-conducted analysis of longitudinal data exploring gestational age and development. The analysis is conducted across several developmental domains: communication, problem solving, personal-social, fine motor, and gross motor. The work helps contribute to a growing understanding of the both the risks posed by late preterm and early preterm birth as well as the vulnerability to delay that can occur across early childhood. The findings also offer an indication of where the focus in domain specific intervention might take.

**Author response:** We thank Reviewer #1 for their comments.

**Reviewer #2:**

1. Thank you for the opportunity to review this interesting work, looking at developmental trajectories in children according to gestational age at birth, using the different domains assessed in the Ages and Stages questionnaire at points between 1 and 5 years of age. This is a very important area of work, given the mounting evidence that outcomes in early childhood are inversely proportional to gestational age. It is now clear that gestational age is a continuum, rather than a dichotomy, and so it is increasingly important to know more detail about the types of problems encountered by these children, and what happens to them as they get older, in order to determine whether, how and when to target interventions to support their health, educational and developmental needs.

**Author response:** We would like to thank Reviewer #2 for this comment.

2. The large tables and limited explanatory text are somewhat challenging for readers who are unfamiliar with the methodology used. Could they perhaps be simplified, or explained more fully in the text? Figure 2 provides a more accessible diagramatic representation that is helpful to understanding of the complexities of the results. Overall, the results appear to be consistent with other work in the field and show a relationship between gestational age and outcomes. This paper may go some way to clarifying the particular issues faced by children born late preterm and determining where, within the wide spectrum of late preterm birth, the main issues lie.

**Author response:** We added additional text in the Results section under “Description of trajectory groups by model” to explain the tables.

3. The authors have appropriately highlighted some limitations to their work. The limitations of most concern is the relatively small numbers falling within some of the groups, which invites the question of whether similar results would be seen in a much larger cohort, or whether this might demonstrate more consistency in the children's developmental trajectories. The authors state that the area studied has a relatively affluent population, and rightly suggest that this may affect the results, since it is well-accepted that trajectories may be modified considerably depending on the socioeconomic status of the family. These results may therefore not be applicable to a less well-off or educated population.

**Author response:** We would like to thank Reviewer #2 for their thoughtful comments. As Reviewer #2 commented, the relatively small numbers within some of the groups invite the question of whether similar results would be seen in a larger cohort or whether this (the small sample size within the trajectory group) might demonstrate more consistency in the children's developmental trajectories. During model specification, we specified the base number of groups using the Bayesian Information Criterion and the Akaike Information Criterion values, but we also visually inspected these groups by graphing the mean values and confidence intervals throughout the model selection process to ensure that we were not separating or combining distinct groups based solely on those criterion values. If repeating this process in a larger cohort we may see similar results if applying this same method, as the sample sizes within the high emerging or high transient groups within the present study are similar to that seen within the persistent and emerging groups reported by Hornman et al in 2017 where they investigated the stability of ASQ-3 scores at ages 4 and 5 within the Longitudinal Preterm Outcome Project (LOLLIPOP) [cohort study](https://www.sciencedirect.com/topics/medicine-and-dentistry/cohort-analysis). However, as we cannot increase the cohort sample size we cannot say for certain. Therefore, we have expanded the methods section to provide more information on model selection, and the limitations section to include more discussion on the role of sample size.

In response to the second comment, “These results may therefore not be applicable to a less well-off or educated population,” we have expanded the limitations section on how this affluence biases the presented results.

**Reviewer #3:**

1. Thank you for asking me to review this manuscript which discusses an interesting topic. The paper is well-written. However, I have some issues with the conceptualisation of the analysis and therefore the practical relevance to practice. I would have thought that based on the ‘introduction’ the authors would pursue the analysis in the line of demonstrating how risks / trajectories change based on late preterm, PT (≥34 to < 37) and early term, ET (≥37 to <39 weeks) referent to full-term (defined as ≥39 to <41). Surprising, this was not done. Merely indicating that the trajectories of developmental delays with decreasing gestation age doesn’t add much value to knowledge. Can we categorise the GA as exposure? Is it possible to know if late PT and early term babies have differing risks / trajectories to FT, and can authors also directly compare the risks between PT and ET? I think that would be more helpful for practice.

**Author response:** Thank you so much for your comment. We agree that the late preterm and early term infants are not the same as full-term born infants, however even within these broad categories our study shows that there are important differences. We feel that by analysing gestational age as a continuous variable in weeks is potentially more relevant for clinical practice, as it allows clinicians to provide targeted information for an individualized care plan for their patients using specific gestational ages.
