# Supplementary material for: Early childhood trajectories of domain-specific developmental delay and gestational age at birth: An analysis of the All Our Families cohort
Source: PLoS One. 2023 Dec 27;18(12):e0294522. doi: 10.1371/journal.pone.0294522 (PMC10752539; doi:10.1371/journal.pone.0294522)
Supplement: Attachment — Submitted filename: Response to Reviewer.docx [file pone.0294522.s002.docx]

**Response to Reviewers**

**Reviewer #3:** It appears there is a fundamental issue with the sample size, hence the reluctance of authors not to examine how the specific timings of birth – late preterm, PT (≥34 to < 37), and early term, ET (≥37 to <39 weeks), referent to full-term (defined as ≥39 to <41) relates with developmental trajectory. More so, that was their line of argument in the introduction. If the categorisation is irrelevant to clinical practice, then it shouldn’t be in the introduction. In addition, the third point raised by Review #3 was not properly addressed in the limitation section – generalisability issues. I think we should be clear if the current findings are generalisable.

This is a well-designed study, however, there seems to be hidden fundamental issue with the sample size that brings into question the generalisability of the findings and relevance to practice. This manuscript is likely to generate a lot of queries with readers.

**Response:**

Thank you for the comments. We respectfully disagree that this is an issue of sample size; the sample sizes according to gestational age categories are displayed in Table 1. Table 1 is stratified by gestational age categories to inform readers of the distribution of the sample characteristics across the range of gestational ages, and the sample sizes within each of the gestational age categories provide readers with additional information on the distribution of the exposure than if only reporting a mean ± SD. The planned analysis chose to include gestational age as a measured/continuous variable a priori for the following reasons:

1) Gestational age is a biological continuum, and while we acknowledge that categories are used for clinical decision-making, these categories are somewhat arbitrary as an infant born at 36 6/7 weeks of gestation compared to an infant born at 37 0/7 are categorized into two separate groups and are clinically treated differently, though their births may only be minutes apart.
2) Apgar scores, a routinely used measure of health status of newborns, are divided into clinically accepted and widely used categories; similar to gestational age. However, studies show increased risks of neonatal morbidity and mortality with lower Apgar scores among infants within the same Apgar category.[1-4] The present analysis, using gestational age by week, speaks to similar issues with using gestational age as a categorical variable.
3) This paper provides new information, where many studies assess differences between gestational age categories, these results show that the risk of developmental delay increases incrementally with each week of gestational age. A literature review identified 8 systematic reviews for LPT births, 2 for ET terms, and 2 more for LPT and ET combined.[5-16] The authors of those reviews state that drawing conclusions from the aggregated data was difficult due to limitations such as lack of uniformity in reporting gestational age categories.[15] The present study's findings add to the body of evidence by reporting results per week of gestational age to reduce those identified limitations.

While we had referred to gestational age categories within the introduction to describe the current body of evidence (which predominantly reports estimates by gestational age categories), we have made minor changes to the introduction and methods to reflect our a priori planned analysis using gestational age as a continuous variable.

With regards to the Reviewer’s comment, “the third point raised by Review #3 was not properly addressed in the limitation section”, we are unclear as to which comment the Reviewer is referring to as Reviewer #3 only had one comment in the last revision. If you could provide clarification, we will respond accordingly.

References

1. 1.	Persson M, Razaz N, Tedroff K, Joseph KS, Cnattingius S. Five and 10 minute Apgar scores and risks of cerebral palsy and epilepsy: population based cohort study in Sweden. Bmj. 2018;360:k207. Epub 20180207. doi: 10.1136/bmj.k207. PubMed PMID: 29437691; PubMed Central PMCID: PMCPMC5802319.
2. 2.	Razaz N, Boyce WT, Brownell M, Jutte D, Tremlett H, Marrie RA, et al. Five-minute Apgar score as a marker for developmental vulnerability at 5 years of age. Arch Dis Child Fetal Neonatal Ed. 2016;101(2):F114-20. Epub 20150717. doi: 10.1136/archdischild-2015-308458. PubMed PMID: 26187935; PubMed Central PMCID: PMCPMC4789716.
3. 3.	Razaz N, Cnattingius S, Joseph KS. Association between Apgar scores of 7 to 9 and neonatal mortality and morbidity: population based cohort study of term infants in Sweden. Bmj. 2019;365:l1656. Epub 20190507. doi: 10.1136/bmj.l1656. PubMed PMID: 31064770; PubMed Central PMCID: PMCPMC6503461.
4. 4.	Razaz N, Cnattingius S, Persson M, Tedroff K, Lisonkova S, Joseph KS. One-minute and five-minute Apgar scores and child developmental health at 5 years of age: a population-based cohort study in British Columbia, Canada. BMJ Open. 2019;9(5):e027655. Epub 20190509. doi: 10.1136/bmjopen-2018-027655. PubMed PMID: 31072859; PubMed Central PMCID: PMCPMC6528022.
5. 5.	Boswinkel V, Nijboer-Oosterveld J, Nijholt IM, Edens MA, Mulder-de Tollenaer SM, Boomsma MF, et al. A systematic review on brain injury and altered brain development in moderate-late preterm infants. Early Hum Dev. 2020;148:105094. Epub 2020/07/28. doi: 10.1016/j.earlhumdev.2020.105094. PubMed PMID: 32711341.
6. 6.	Fernández de Gamarra-Oca L, Ojeda N, Gómez-Gastiasoro A, Peña J, Ibarretxe-Bilbao N, García-Guerrero MA, et al. Long-Term Neurodevelopmental Outcomes after Moderate and Late Preterm Birth: A Systematic Review. J Pediatr. 2021;237:168-76.e11. Epub 20210624. doi: 10.1016/j.jpeds.2021.06.004. PubMed PMID: 34171360.
7. 7.	Machado Júnior LC, Passini Júnior R, Rodrigues Machado Rosa I. Late prematurity: a systematic review. J Pediatr (Rio J). 2014;90(3):221-31. Epub 2014/02/11. doi: 10.1016/j.jped.2013.08.012. PubMed PMID: 24508009.
8. 8.	Martínez-Nadal S, Bosch L. Cognitive and Learning Outcomes in Late Preterm Infants at School Age: A Systematic Review. Int J Environ Res Public Health. 2020;18(1). Epub 2020/12/31. doi: 10.3390/ijerph18010074. PubMed PMID: 33374182; PubMed Central PMCID: PMCPMC7795904.
9. 9.	McGowan JE, Alderdice FA, Holmes VA, Johnston L. Early Childhood Development of Late-Preterm Infants: A Systematic Review. Pediatrics. 2011;127(6):1111. doi: 10.1542/peds.2010-2257.
10. 10.	Srinivasjois R, Silva D, Maina K. Preschool outcomes of late preterm infants-a systematic review. 2014.
11. 11.	Teune MJ, Bakhuizen S, Gyamfi Bannerman C, Opmeer BC, van Kaam AH, van Wassenaer AG, et al. A systematic review of severe morbidity in infants born late preterm. Am J Obstet Gynecol. 2011;205(4):374.e1-9. Epub 2011/08/26. doi: 10.1016/j.ajog.2011.07.015. PubMed PMID: 21864824.
12. 12.	Tripathi T, Dusing SC. Long-term neurodevelopmental outcomes of infants born late preterm: a systematic review. Research and Reports in Neonatology. 2015;5:91-111.
13. 13.	Dong Y, Chen SJ, Yu JL. A systematic review and meta-analysis of long-term development of early term infants. Neonatology. 2012;102(3):212-21. Epub 2012/07/21. doi: 10.1159/000338099. PubMed PMID: 22814228.
14. 14.	Nielsen TM, Pedersen MV, Milidou I, Glavind J, Henriksen TB. Long‐term cognition and behavior in children born at early term gestation: A systematic review. Acta Obstetricia et Gynecologica Scandinavica. 2019;98(10):1227-34. doi: 10.1111/aogs.13644.
15. 15.	Murray SR, Shenkin SD, McIntosh K, Lim J, Grove B, Pell JP, et al. Long term cognitive outcomes of early term (37-38 weeks) and late preterm (34-36 weeks) births: A systematic review. Wellcome Open Res. 2017;2:101-. doi: 10.12688/wellcomeopenres.12783.1. PubMed PMID: 29387801; PubMed Central PMCID: PMCPMC5721566.
16. 16.	Chan E, Leong P, Malouf R, Quigley MA. Long-term cognitive and school outcomes of late-preterm and early-term births: a systematic review. Child Care Health Dev. 2016;42(3):297-312. Epub 2016/02/11. doi: 10.1111/cch.12320. PubMed PMID: 26860873.
